# The influence of geometric algebra in surgical practice of sleeve gastrectomy-single center experience

**DOI:** 10.1097/MD.0000000000030783

**Published:** 2022-10-28

**Authors:** Gang Yao, Amina Aierken, Tao Li, Xinling Cao, Shadika Apaer, Nuerzhatijaing Anweier, Jing Wu, Xiapukaiti Fulati, Yun-Fei Zhang, Abudushalamu Tuerxunmaimaiti, Tuerhongjiang Tuxun

**Affiliations:** a Department of Liver & Laparoscopic Surgery, Center of Digestive and Vascular Surgery, The First Affiliated Hospital of Xinjiang Medical University, Urumqi, China; b Health Management Institute, Xinjiang Medical University, Urumqi, China.

**Keywords:** appendectomy, laparoscopy, outcome, pregnancy

## Abstract

Laparoscopic surgery could be considered as an art of geometric algebra. However, very little is studied in the context of bariatric surgery. The current study aims to explore the possible influence concept of geometric algebra on the surgical process in the overweight and obese patients in the setting of laparoscopic sleeve gastrectomy (LSG). During the study period, clinical data of subjects who underwent LSG was retrospectively analyzed. Parameters examined include body mass index (BMI), umbilical-xiphoidal interval (U-X) and umbilical-fundus (U-F) interval. In this study, LSG was performed via central view approach (C) and left view approach (L). In both groups, the body surface projection points of viewing hole (V), main and accessory operating holes (O_1_ and O_2_) and surface display of fundus (F) were connected to form a geometric figure. The accessibility of the surgical instrument into the fundus, the need for elongated instruments and related intra- and post-operative parameters were noted. The overweight and obese subjects showed a significant increased U-X and U-F interval compared to the non-obese subjects. The length of both U-X and U-F interval were correlated with the BMI. The geometric figure is quite different between L and C approach with significant increase of area of quadrangle. Significant longer O_1_-F, O_2_-F and V-F interval was calculated in C approach of patients and thus the elongated instruments were frequently required. The integration of the concept geometric algebra with the proper selection of troca may provide a better surgical experience and smooth surgical process.

## 1. Introduction

Despite the existence of poverty in some regions, with development of economic status and change of lifestyle, the incidence of obesity around the world is increasing rapidly.^[1]^ Obesity not only becomes a social problem but also a critical health issue which threatens peoples’ lives. It is widely accepted that, obesity is closely associated with metabolic disorder diseases including diabetes mellitus type 2 (DM2), hypertension, hyperlipemia, hyperuricemia, polycystic ovarian syndrome and obstructive sleep apnea hypopnea syndrome (OSAS).^[2]^ Over half a century, bariatric surgery has become one of the indispensable modalities for obesity control. Surgical community has gradually formed its standards and criteria with exponential growth of bariatric operation performed.^[3]^ Increasing evidence claimed the short- and long-term clinical efficacy of bariatric surgery on morbid obesity and concomitant hypertension and DM2.^[4]^ According to data of the 4th International Federation for the Surgery of Obesity (IFSO) Global Registry, 190177 cases of bariatric surgery have been reported worldwide.^[5]^ Of note, the actual number of patients underwent bariatric surgery would be higher than the report. Surgical procedure for morbid obesity evolved rapidly from vertical gastric banding to sleeve gastrectomy and gastric bypass. Besides, several modified surgical approaches have been proposed for better clinical outcome.^[6]^

Amongst the various surgical procedures, sleeve gastrectomy is relatively easy to perform and associated with less complication nut nearly equal postoperative outcome compared to gastric bypass. According to the IFSO registry, the number of patients who received sleeve gastrectomy have surpassed the patients with gastric bypass and become most welcomed surgical approach both for surgeons and patients.^[5]^ In United States, sleeve gastrectomy has been the most commonly performed bariatric procedure and accounts for 61.4% of all bariatric operations.^[7]^ Similarly, a recent large-scale analysis showed sleeve gastrectomy accounts for nearly 73% of all registered bariatric operations in China.^[8]^

Despite being technically easier, sleeve gastrectomy still imposes a great challenge for bariatric surgeons, especially encountering to patients with higher body mass index (BMI). Several factors including the skill of surgeons, surgical instruments and patient’s status may influence safety and course of the operation. Among them, patients’ parameters including BMI, abdominal wall thickness and umbilical-xiphoid interval are the critical for successful operations. In current study, we are aiming to focus on parameters, from the aspect of geometric algebra that may influence the practice of laparoscopic sleeve gastrectomy (LSG).

## 2. Materials and Methods

### 2.1. Study design and data set

A total number of 150 non-obese volunteers and 128 obese patients underwent sleeve gastrectomy were enrolled into this study during the period of January 2015 to December 2020.

For LSG, patients with a BMI over 32.5 at least with one associated comorbidity were included. All the obese patients were assessed by a multidisciplinary team (MDT) including bariatric surgeons, anesthesiologist, gastroenterologist, gynecologists and nutritionists. Blood routine test, liver function, kidney function, fasting glucose, glycated hemoglobin (HbA1c), insulin, blood urea nitrogen (BUN), creatinine, electrolytes, lipid panel, thyroid-stimulating hormone (TSH), chest X-ray, electrocardiogram, echocardiogram, thyroid ultrasound, abdominal ultrasound, thoraco-abdominal-pelvic CT scan, and upper gastrointestinal endoscopy (UGE) were routinely carried out. The design and conduction of current study were approved by the ethical committee of authors’ institutions. Informed consents were obtained from all participants and/or their legal custodies. Patients with insufficient clinical data and underwent clinical procedures other than LSG were excluded.

### 2.2. Mathematical parameters

Mathematical parameters were measured and recorded on the first day of admission for all participants. The measured parameters include BMI, umbilical-xiphoid process interval (U-X), umbilical-fundus interval (U-F), abdominal wall thickness, and abdominal anteroposterior diameter. For the measurement of intervals, the surface projection of umbilical, xiphoid process and fundus of the stomach were marked prior surgery and recorded, respectively. The abdominal wall thickness was measured based upon the CT scan by using picture archiving and communicating system (PACS).

### 2.3. Clinical parameters

Baseline information and operative parameters were recorded and analyzed for obese patients who underwent LSG. Preoperative baseline information included age, gender, waistline, hipline, waist-hip ratio. The existence of concomitant diseases including DM2, hypertension, hyperlipemia, hyperuricemia, OSAS and polycystic ovary syndrome were recorded if any. Meanwhile, operative parameters included operative time, blood loss and difficulty for access and dissect gastric fundus, fundus dissection time, suture tine, post-operative hospital stays, morbidity and 3-year excess weight loss (EWL). The %EWL was calculated using formula (weight loss/baseline excess weight) × 100, where excess weight = initial weight – ideal weight (ideal BMI = 23 kg/m^2^).

### 2.4. Surgical procedure

**Figure 1.**
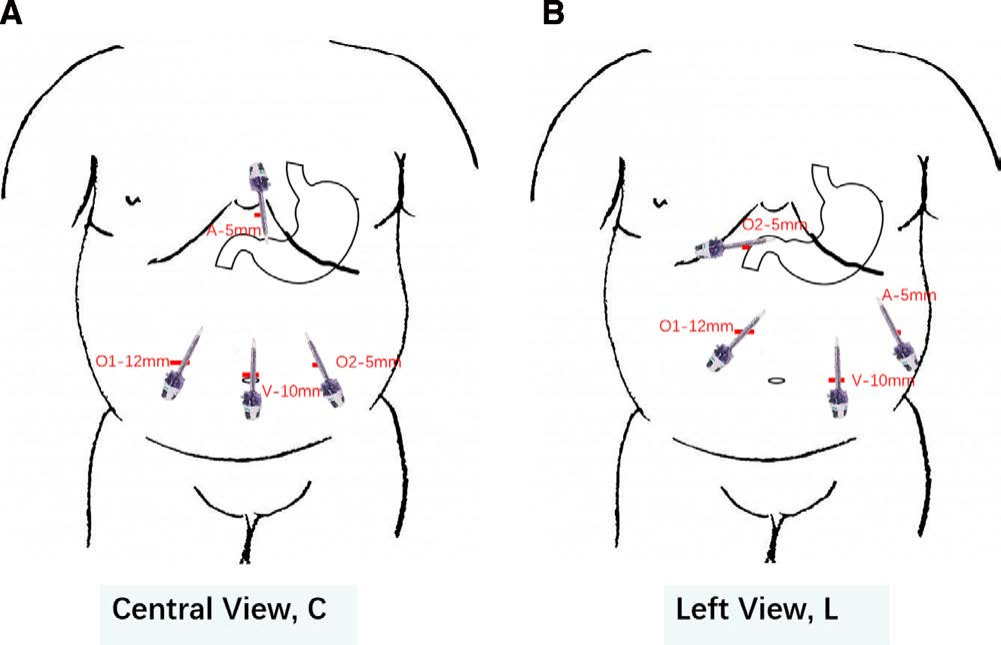
The troca position sites in patients with C and L approach. (A) Troca position in C approach and (B) Troca position in L approach. A = assistance hole, F = fundus; O1 = operating hole 1, O2 = operating hole 2, V = viewing hole.

Patients were operated with a four-port laparoscopic vertical gastrectomy technique in supine position. The operated patients were divided into two groups including central view approach (C) and left view approach (L) based upon their unique troca position, especially for the viewing hole. The detailed the troca positions were shown in Figure 1. The sleeve was performed from antrum to the angle of Hiss, starting at 5 cm from pylorus and with a 36f boogie calibration. Left hiatal crus were always exposed in order to find and repair any possible hiatal hernia. Reinforced suture to stapler line was routinely performed by using 2-0 V-lock thread. All procedures were by the same team in authors’ institution. The difficulty to access and dissect the fundus was assessed by chief surgeons by scoring the accessibility and feasibility from 1 to 5 points. Score 1 represents very difficult, score 2 difficult, score 3 not difficult, score 4 easy and score 5 very easy.

### 2.5. Statistics

Numeric data were presented as median + SD, the SPSS statistical package (Chicago, IL) was used. The normal distribution continuous variables were assessed with Student *t* test. A *P* value < .05 was defined as statistical significance.

## 3. Results

### 3.1. Baseline information of all subjects

The baseline information of studied subjects including 150 non-obese and 128 obese patients was tabulated in Table 1. The BMI, U-X interval, U-F interval, abdominal thickness and abdominal anteroposterior diameters were significantly higher in obese patients.

**Table 1:** Geometrical parameters of 150 non-obese and 128 obese subjects.

	Non-obese (n = 150)	Obese (n = 128)	*t*	*P*
Age (yr)	36.3 ± 3.11	36.11 ± 3.80	0.696	>.05
BMI (kg/m^2^)	20.31 ± 3.72	35.56 ± 3.11	36.71	<.0001
U-X interval (cm)	13.39 ± 2.03	19.12 ± 3.44	17.19	<.0001
U-F interval (cm)	23.08 ± 2.26	29.64 ± 4.03	17.04	<.0001
Abdominal thickness (cm)	2.51 ± 1.01	5.07 ± 2.31	12.27	<.0001
Abdominal anteroposterior diameter (cm)	25.44 ± 4.18	35.62 ± 6.22	16.21	<.0001

BMI = body mass index.

### 3.2. Baseline information of obese patients

The baseline information of obese patients who underwent LSG was given in Table 2. There was no statistical significance regarding the sex, age, BMI, waistline, hipline and comorbidities between central approach (C) group and left approach (L) group.

**Table 2:** Baseline information of 128 obese patients undergoing sleeve gastrectomy.

	C approach (n = 72)	L approach (n = 56)		*P*
Sex	76	52		
Male	22	18	0.462	.562
Female	54	34		
Age (yrs)	30 ± 4	31 ± 6	1.129	.2612
BMI (kg/m^2^)	37.8 ± 5.4	38.1 ± 5.6	0.3068	.759
Waistline (cm)	114.6 ± 13	115.3 ± 12.6	0.3063	.7599
Hipline (cm)	119.7 ± 11	122 ± 10.7	1.188	.2372
Waist/hip ratio	0.97 ± 0.14	0.96 ± 0.12	0.4263	.6706
Comorbidity				
Diabetes	57	36	3.511	.061
Hypertension	28	17	1.006	.316
Hyperlipidemia	55	38	1.154	.283
Hyperuricemia	42	28	0.883	.347
Moderate/severe OSAS	59	38	3.406	.065
PCOS	17	9	1.106	.293

BMI = body mass index, OSAS = obstructive sleep apnea hypopnea syndrome, PCOS = polycystic ovary syndrome.

### 3.3. Mathematical parameters in two groups

**Figure 2.**
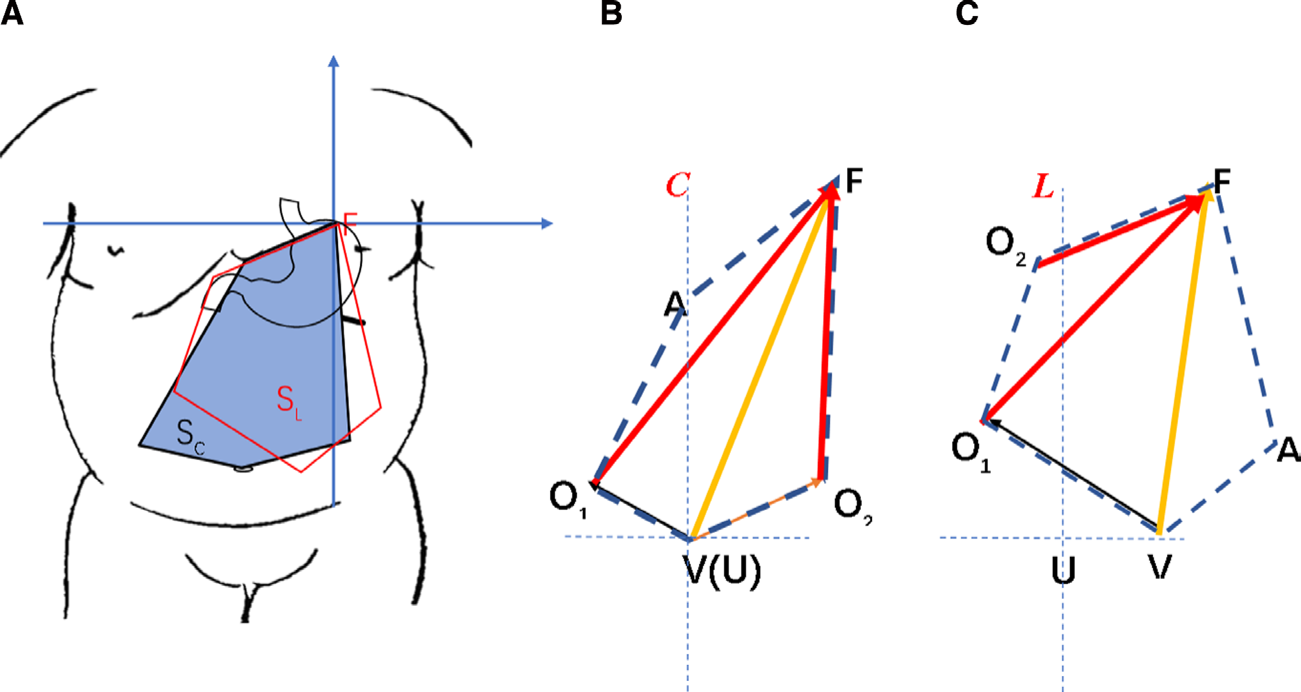
Geometrical figure of obese patients underwent LSG. (A) area of operating field in C and L group, (B) geometrical figure in C group and (C) geometrical figure in L group. A = assistance hole, F = fundus, O1 = operating hole 1, O2 = operating hole 2, U = umbilical, V = viewing hole.

The marking points of surface projections of viewing hole (V), main operating hole (O1 and O2), accessory hole (A) and fundus were connected to form a geometric figure. The area of geometric shape in central approach and left approach are S_C_ and S_L_. Sc is significantly bigger than S_L_ as shown in Figure 2A. Besides, the distance between O1-F, O2- F and V-F were significantly longer in C group compared to L group as shown in Figure 2B and C.

### 3.4. Operative parameters in two groups

LSG in all obese patients was successfully performed with no need for conversion to open surgery. No statistical significance was found regarding operative time, blood loss, post-operative hospital stays, morbidity and 3-year EWL% between two groups. However, the fundus dissection time and suture time were longer with fundus exposure difficulty score higher in group C compared to group L (Table 3).

**Table 3:** Operative parameters in patients with C and L approach.

Parameters	C approach (n = 72)	L approach (n = 56)	*t*	*P*
Operative time (min)	120 ± 19	122 ± 20	0.5773	.5647
Blood loss (mL)	50 ± 15	46 ± 11	1.675	.0964
Fundus dissection time (min)	30 ± 12	22 ± 9	4.16	<.0001
Fundus exposure difficulty score	3.2 ± 0.31	4 ± 0.27	15.31	<.0001
Suture time (min)	12.3 ± 3.42	15.8 ± 5.51	4.41	<.0001
Post-operative hospital stays (d)	3.1 ± 1.20	3.2 ± 1.11	0.4832	.6298
Morbidity				
Bleeding	1	0		1.000
Gastric fistula	0	0		
Surgical site infection	0	0		
Electrolyte disorder	0	0		
3-year EWL%	60 ± 18	58 ± 20	0.5939	.5536

Fundus exposure difficulty and accessibility is scored by the chief surgeons based upon the difficulty and satisfaction after surgery. Score 1: Very difficult; Score 2: Difficult: Score 3: Not difficult: Score 2: Easy; Score 1: Satisfied.

EWL = excess weight loss.

## 4. Discussion

This is the first study, to the best of our knowledge that report the bariatric surgery from the mathematical perspectives. Our results demonstrated that proper troca positioning based upon geometrical parameters may provide better surgical experience and outcome.

Over the half century, bariatric surgery has developed in rocket speed with the increasing number of bariatric surgeons and obese patients who underwent surgical interventions.^[9]^ To date, LSG is the mostly performed surgical operation for obese patients around world with nearly 92% of all bariatric surgery performed in China according to the annual report 2020.^[10]^ Surgical societies issued a standard operating step, guidelines regarding the sleeve gastrectomy in order to standardize the surgical practice and to reduce the postoperative morbidity as well as mortality.^[11]^

The safety issue is the priority and therefore every single LSG should be critically planned and performed. There are several influencing factors from patient (BMI, comorbidities, abdominal wall thickness), surgeon (technical excellence, volume of center) and instrumental perspective (camera, elongated instruments, etc.), respectively. Surgical accessibility, exposure and proper management of short gastric vessels to free the fundus of stomach is vital importance for successful surgery, therefore, the suitable trocar positing may help surgeons to master the surgical filed and accomplish the surgery in a comfortable way. The unsuitable trocar positioning may result in inadequate exposure to the gastric fundus, difficult dissection of the short gastric vessels and sometimes may lead to uncontrollable bleeding. Some time, the fatty contents of abdominal hurdles surgeons to localize exact bleeding sites and the improper control may result in intra- and/or post-operative bleeding which is the most lethal complication after LSG.^[12]^ Several centers reported the intraoperative bleeding due to inadequate exposure and such a situation was occurred in our center with early experience. Besides, the adequate dissection of fundus is also important to achieve better weight loss and discover possible existence of hiatal hernia.^[13]^

In this study, we connected the trocar points and gastric fundus into a geometrical figure in patients undergoing LSG with two distinct approaches including central view and left view. The C approach was mostly applied by majority of bariatric surgeons, since the trocar positing for main and accessory operating hole are in conformity with the “Triangle” principle.^[14]^ However, with longer U-X and U-F distances, this approach may have difficulty in exposure and dissection of fundus and usually call for the elongated surgical instrument for completion of surgery. When this approach meets with patients with BMI higher than 50, thick abdominal wall and deep anteroposterior trunk diameters, surgical time and surgical difficulty are increased even with the help of assistance to expose the surgical field. In contrast, L approach is associated with shorten distance to the operating field and fastened exposure of the fundus. Moreover, this approach showed no high requirement to the assistance. In our early experience, the time duration of sleeve suture is longer in patients in L approach, with no statistical significance in comparison to C approach. Abdominal wall sickness is an important parameter for both approaches. When the abdominal wall sickness is broader than 8 cm, it poses a great difficulty during the operation by fixing the troca, and therefore the mobility of the instruments is limited. In our center, preoperative abdominal sickness assessment if mandate, and the elongated troca could be optional in patients with super sickness.

There are some limitations should be addressed. First, this is a retrospective single center experience and may not be suitable for other centers. However, this is the first study reporting LSG from the point of geometric algebra. Secondly, relatively small number of patients are enrolled in this study and further assessment is strongly advocated.

## 5. Conclusion

LSG could be considered as an art of geometric algebra, preoperative precise assessment of patients and proper troca positioning are the key for successful surgery. Left approach seems to provide better exposure and comfortable experience.

## Author contributions

**Conceptualization:** Gang Yao, Tuerhongjiang Tuxun.

**Data curation:** Amina Aierken, Gang Yao.

**Formal analysis:** Tuerhongjiang Tuxun, Xiapukaiti Fulati.

**Funding acquisition:** Tuerhongjiang Tuxun.

**Investigation:** Tao Li, Tuerhongjiang Tuxun, Xinling Cao.

**Methodology:** Amina Aierken, Jing Wu, Nuerzhatijaing Anweier, Shadika Apaer, Xiapukaiti Fulati, Yun-Fei Zhang.

**Resources:** Tao Li.

**Writing – original draft:** Abudushalamu Tuerxunmaimaiti, Tuerhongjiang Tuxun.

**Writing – review & editing:** Tuerhongjiang Tuxun.
